# Author Correction: Recent evolution of a TET-controlled and DPPA3/STELLA-driven pathway of passive DNA demethylation in mammals

**DOI:** 10.1038/s41467-020-20453-0

**Published:** 2020-12-17

**Authors:** Christopher B. Mulholland, Atsuya Nishiyama, Joel Ryan, Ryohei Nakamura, Merve Yiğit, Ivo M. Glück, Carina Trummer, Weihua Qin, Michael D. Bartoschek, Franziska R. Traube, Edris Parsa, Enes Ugur, Miha Modic, Aishwarya Acharya, Paul Stolz, Christoph Ziegenhain, Michael Wierer, Wolfgang Enard, Thomas Carell, Don C. Lamb, Hiroyuki Takeda, Makoto Nakanishi, Sebastian Bultmann, Heinrich Leonhardt

**Affiliations:** 1 Department of Biology II and Center for Integrated Protein Science Munich (CIPSM), Human Biology and BioImaging, Ludwig-Maximilians-Universität München, Planegg-Martinsried, Germany; 2 Division of Cancer Cell Biology, The Institute of Medical Science, The University of Tokyo, 4-6-1 Shirokanedai, Minato-ku, Tokyo, 108-8639 Japan; 3 Department of Biological Sciences, Graduate School of Science, The University of Tokyo, 7-3-1 Hongo, Bunkyo-ku, Tokyo, 113-0033 Japan; 4 Physical Chemistry, Department of Chemistry, Center for Nanoscience, Nanosystems Initiative Munich and Center for Integrated Protein Science Munich, Ludwig-Maximilians-Universität München, Munich, Germany; 5 Center for Integrated Protein Science (CIPSM) at the Department of Chemistry, Ludwig-Maximilians-Universität München, Munich, Germany; 6 Department of Proteomics and Signal Transduction, Max Planck Institute for Biochemistry, Am Klopferspitz 18, 82152 Martinsried, Germany; 7 The Francis Crick Institute and UCL Queen Square Institute of Neurology, London, UK; 8 Department of Biology II, Anthropology and Human Genomics, Ludwig-Maximilians-Universität München, Planegg-Martinsried, Germany

**Keywords:** Pluripotency, Reprogramming, Epigenetics, DNA methylation, Epigenomics

Correction to: *Nature Communications* 10.1038/s41467-020-19603-1, published online 24 November 2020.

The original version of this Article contained an error in the spelling of the author Makoto Nakanishi, which was incorrectly given as Makoto Nakanashi. This has now been corrected in both the PDF and HTML versions of the Article.

