# Supplementary material for: Recent evolution of a TET-controlled and DPPA3/STELLA-driven pathway of passive DNA demethylation in mammals
Source: Nat Commun. 2020 Nov 24;11:5972. doi: 10.1038/s41467-020-19603-1 (PMC7686362; doi:10.1038/s41467-020-19603-1)
Supplement: Supplementary file 1 — Supplementary Information [file 41467_2020_19603_MOESM1_ESM.pdf]

SUPPLEMENTARY FIGURES for:

Recent evolution of a TET-controlled and DPPA3/STELLA-driven pathway of passive demethylation in mammals

Authors:

Christopher B. Mulholland[1], Atsuya Nishiyama[2†], Joel Ryan[1†], Ryohei Nakamura[3], Merve Yiğit[1], Ivo M. Glück[4], Carina Trummer[1], Weihua Qin[1], Michael D. Bartoschek[1], Franziska R. Traube[5], Edris Parsa[5], Enes Ugur[1,8], Miha Modic[7], Aishwarya Acharya[1], Paul Stolz[1], Christoph Ziegenhain[6], Michael Wierer[8], Wolfgang Enard[6], Thomas Carell[5], Don C. Lamb[4], Hiroyuki Takeda[3], Makoto Nakanashi[2], Sebastian Bultmann[*1§] and Heinrich Leonhardt[*1§]

Correspondence: bultmann@bio.lmu.de or h.leonhardt@lmu.de

This PDF includes:

Supplementary Fig. 1
Supplementary Fig. 2
Supplementary Fig. 3
Supplementary Fig. 4
Supplementary Fig. 5
Supplementary Fig. 6
Supplementary Fig. 7
Supplementary Fig. 8
Supplementary Fig. 9

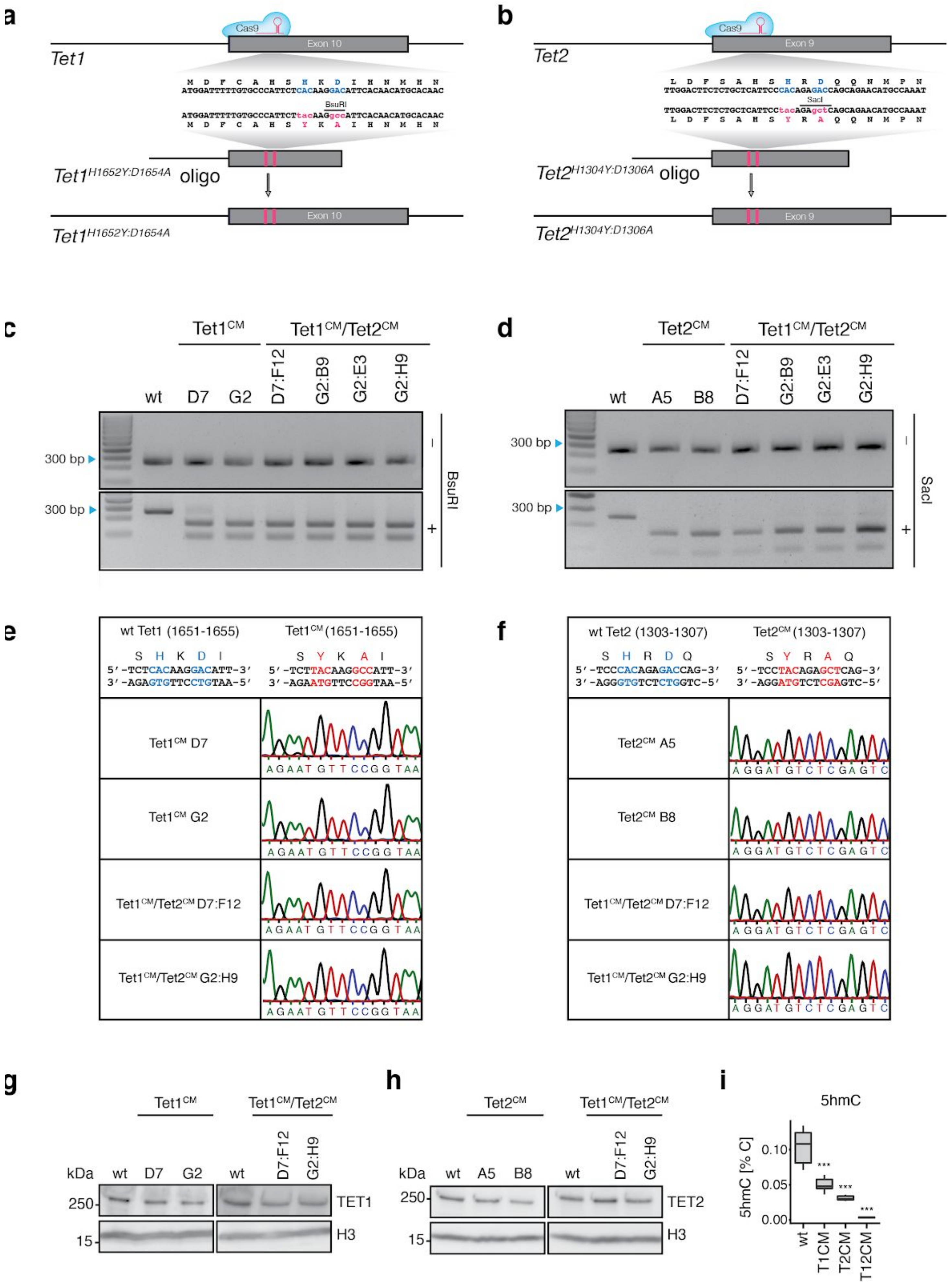
a
Cas9
Exon 10
Tet1
BsuRI
Tet1H1652Y:D1654A oligo
Tet1H1652Y:D1654A
b
Cas9
Exon 9
Tet2
SacI
Tet2H1304Y:D1306A oligo
Tet2H1304Y:D1306A
c
Tet1CM
Tet1CM/Tet2CM
wt
D7
G2
D7:F12
G2:B9
G2:E3
G2:H9
300 bp
300 bp
BsuRI
-
+
d
Tet2CM
Tet1CM/Tet2CM
wt
A5
B8
D7:F12
G2:B9
G2:E3
G2:H9
300 bp
300 bp
SacI
-
+
e
wt Tet1 (1651-1655)
Tet1CM (1651-1655)
S H K D I
5′-TCTCACAAGGACATT-3′
3′-AGAGTGTTCCTGTAA-5′
S Y K A I
5′-TCTTACAAGGCCATT-3′
3′-AGAATGTTCCGGTAA-5′
Tet1CM D7
Tet1CM G2
Tet1CM/Tet2CM D7:F12
Tet1CM/Tet2CM G2:H9
AGAATGTTCCGGTAA
f
wt Tet2 (1303-1307)
Tet2CM (1303-1307)
S H R D Q
5′-TCCCACAGAGACCAG-3′
3′-AGGGTGTCTCTGGTC-5′
S Y R A Q
5′-TCCTACAGAGCTCAG-3′
3′-AGGATGTCTCGAGTC-5′
Tet2CM A5
Tet2CM B8
Tet1CM/Tet2CM D7:F12
Tet1CM/Tet2CM G2:H9
AGGATGTCTCGAGTC
g
Tet1CM
Tet1CM/Tet2CM
kDa
wt
D7
G2
wt
D7:F12
G2:H9
250
15
TET1
H3
h
Tet2CM
Tet1CM/Tet2CM
kDa
wt
A5
B8
wt
D7:F12
G2:H9
250
15
TET2
H3
i
5hmC
5hmC [% C]
0.10
0.05
0.00
***
***
***
wt
T1CM
T2CM
T12CM

Supplementary Figure 1: Generation and characterization of T1CM, T2CM, and T12CM mESCs

a,b, Schematic representation of the CRISPR/Cas9 gene editing strategies used to mutate the catalytic center (HxD iron-chelating motif) of *Tet1* (**a**) and *Tet2* (**b**). gRNA target sequences and restriction enzyme recognition sites for restriction fragment length polymorphism (RFLP) screening are shown (See Supplementary Data 5). **c,d,** Genotyping using RFLP analysis of *Tet1* (**c**) and *Tet2* (**d**) loci. Data in c and d are representative of n = 2 independent experiments. **e,f,** DNA sequencing traces confirming the successful mutation of the HxD motif to YxA in *Tet1* (**e**) and *Tet2* (**f**). The top boxes depict the DNA and corresponding amino acid sequences of the HxD motif before (blue) and after (red) gene editing. **g,h,** Immunoblot detection of endogenous TET1 (**g**) and TET2 (**h**) protein levels in T1CM, T2CM, and T12CM mESC. Data in g and h are representative of n = 3 independent experiments. **i,** DNA modification levels as percentage of total cytosines measured in wt (n = 24), T1CM (n = 8), T2CM (n = 12), and T12CM (n = 11) mESC biological replicates. Depicted are mean values ± standard deviation; ***P < 2e-16 to wt as determined using a one-way ANOVA followed by a post-hoc Tukey HSD test. Source data are provided as a Source Data file.

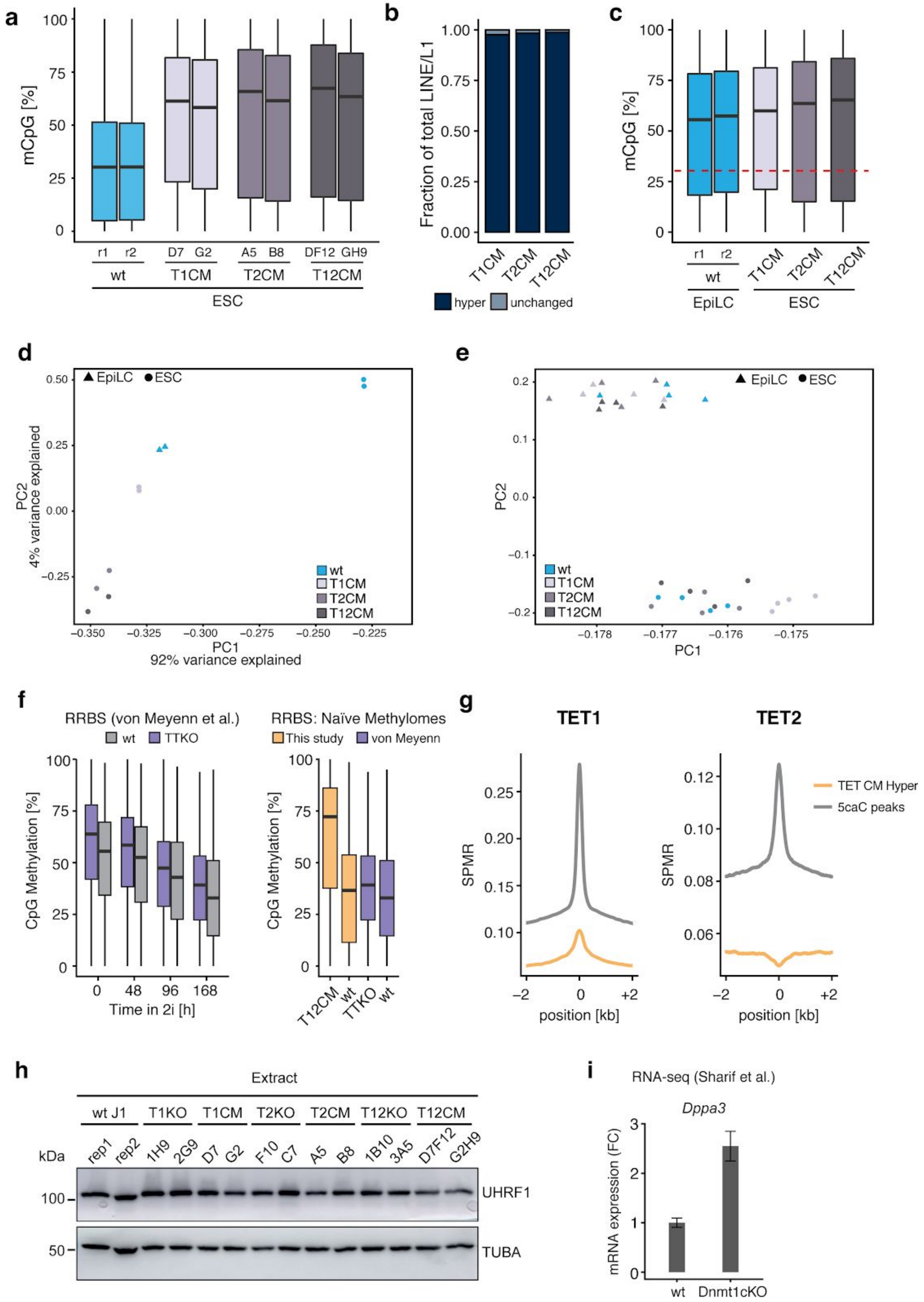

a
mCpG [%]
r1 r2 D7 G2 A5 B8 DF12 GH9
wt T1CM T2CM T12CM
ESC
b
Fraction of total LINE/L1
T1CM T2CM T12CM
hyper unchanged
c
mCpG [%]
r1 r2 T1CM T2CM T12CM
wt
EpiLC ESC
d
EpiLC ESC
PC2 4% variance explained
PC1 92% variance explained
wt T1CM T2CM T12CM
e
EpiLC ESC
PC2
PC1
wt T1CM T2CM T12CM
f
RRBS (von Meyenn et al.)
wt TTKO
CpG Methylation [%]
Time in 2i [h]
RRBS: Naïve Methylomes
This study von Meyenn
T12CM wt TTKO wt
g
TET1
TET2
SPMR
position [kb]
TET CM Hyper
5caC peaks
h
Extract
wt J1 T1KO T1CM T2KO T2CM T12KO T12CM
kDa
rep1 rep2 1H9 2G9 D7 G2 F10 C7 A5 B8 1B10 3A5 D7F12 G2H9
UHRF1
TUBA
i
RNA-seq (Sharif et al.)
Dppa3
mRNA expression (FC)
wt Dnmt1cKO

Supplementary Figure 2: Methylome and transcriptome analysis of T1CM, T2CM, and T12CM ESCs

a, Percentage of total 5mC as measured by RRBS in all analyzed wt, T1, T2, and T12 CM ESC replicates from n = 2 independent cell lines per genotype. **b,** Relative proportion of DNA hypermethylation (q value < 0.05; absolute methylation difference > 20%) at LINE1/L1 elements in T1CM, T2CM, and T12CM ESCs compared to wt ESCs. For each genotype, n = 2 independent clones. **c,** Loss of TET catalytic activity in ESCs results in similar or higher DNA methylation levels than in wt EpiLCs. Comparison of the total CpG DNA methylation (5mCpG) as measured by RRBS between individual wt EpiLC samples (n = 1 for each depicted cell line) and T1CM, T2CM, and T12CM ESCs (n = 2 independent cell lines per genotype). Dashed red line indicates the median mCpG methylation in wt EpiLCs. **d,** Principal component (PC) analysis of RRBS data from wt, T1CM, T2CM, and T12CM ESCs and wt EpiLCs. **e,** PC analysis of RNA-seq data from wt, T1CM, T2CM, and T12CM ESCs during EpiLC differentiation. **f**, Boxplots comparing total 5mC levels in Tet catalytic mutants (T12CM) and Tet triple (TET1, TET2, TET3) knockout ESCs (TTKO)[1] based on RRBS measurements from n = 2 biological replicates per genotype. **g,** TET1[2] and TET2[3] occupancy over 1 kb tiles hypermethylated (orange) in T1CM and T2CM ESCs or regions of 5caC enrichment (grey)[4]. (SPMR: Signal per million reads). **h,** UHRF1 protein levels are unaffected by inactivation or loss of TET proteins. Whole-cell extracts from wt J1, TET catalytic mutant (T1CM, T2CM, and T12CM) and TET knockout (*Tet1* KO (T1KO), *Tet2* KO (T2KO), and *Tet1/2* KO (T12KO))[5] ESCs analyzed by immunoblot detection using the indicated antibodies. Immunoblot depicts *n* = 2 biological replicates (wt J1: rep1 and rep2) or independent clones (TET CM and TET KO ESC lines) for each genotype. **i**, *Dppa3* mRNA levels in wild type and conditional *Dnmt1* knockout cells (Dnmt1cKO) [6]. Error bars indicate the mean ± SD calculated from n = 3 biological replicates. For the boxplots in **a, c,** and **f**, horizontal black lines within boxes represent median values, boxes indicate the upper and lower quartiles, and whiskers extend to the most extreme value within 1.5 x the interquartile range from each hinge. Source data are provided as a Source Data file.

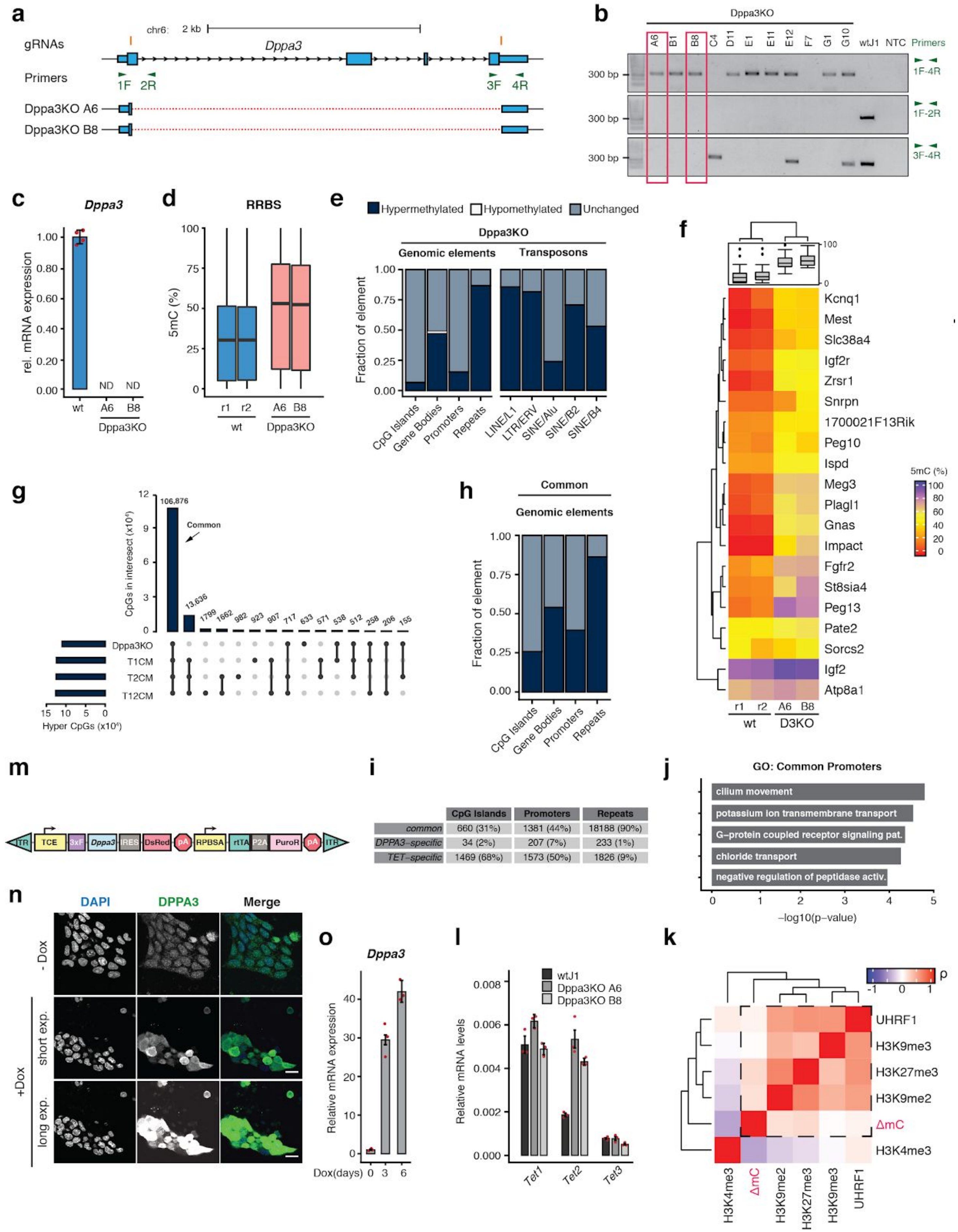

	CpG Islands	Promoters	Repeats
common	660 (31%)	1381 (44%)	18188 (90%)
DPPA3-specific	34 (2%)	207 (7%)	233 (1%)
TET-specific	1469 (68%)	1573 (50%)	1826 (9%)

Supplementary Figure 3: Generation, characterization, and methylome profiling of Dppa3KO ESCs

a, Schematic representation of the CRISPR/Cas9 gene editing strategy used to excise the entire *Dppa3* locus. The position of the two locus-flanking gRNAs are shown in orange. PCR primers for determination of locus removal and zygosity are indicated in green. **b,** Results of PCR using the primers indicated in (**a**). *Dppa3* knockout (Dppa3KO) clones, A6 and B8, chosen for further experiments are indicated by the red boxes. The wild-type (wt) and no template control (NTC) reactions are depicted on the right. Data are representative of n = 2 independent experiments. The sequence alignment of the amplicon generated using primers 1 and 4 for Dppa3KO clones A6 and B8 is provided in the lower portion of (**a**) with solid boxes and the dashed lines representing successfully aligned sequences and genomic sequences not found in the sequenced amplicons respectively. **c,** *Dppa3* transcript levels of the two Dppa3KO clones A6 and B8 are depicted relative to mRNA levels in wt J1 ESCs after normalization to *Gapdh*. Error bars indicate mean ± SD calculated from technical triplicate reactions from *n* = 4 biological replicates. N.D., expression not detectable. **d,** Percentage of total 5mC as measured by RRBS in wt and Dppa3KO ESC replicates (n = 2 independent cell lines per genotype). Horizontal black lines within boxes represent median values, boxes indicate the upper and lower quartiles, and whiskers extend to the most extreme value within 1.5 x the interquartile range from each hinge. **e,** Relative proportion of DNA hypermethylation occurring at each genomic element or retrotransposon class in Dppa3KO ESCs. **f**, Heatmap depicting DNA methylation levels over ICRs in wild type and Dppa3KO ESCs. **g,** Upset plot depicting the overlap of hypermethylated CgGs between DPPAKO, T1CM, T2CM, and T12CM ESCs. Vertical bar plots indicate numbers of overlapping CpGs for each intersection. Intersections are indicated by filled circles connected by black lines. Horizontal bar plots indicate total numbers of hypermethylated CpGs for each cell line**. h,** Relative proportion of DNA hypermethylation common to T1CM, T2CM, and T12CM ESCs and Dppa3KO ESCs at each class of genomic element. Dark blue indicates common hypermethylated elements. **i,** Summary of differentially methylated regions either unique to TET mutants (TET-specific) and Dppa3KO ESCs (DPPA3-specific) or shared among TET mutants and Dppa3KO ESCs (common). **j,** Gene ontology (GO) terms associated with promoters specifically dependent on TET activity; adjusted p-values calculated using two-sided Fisher's exact test. **k,** Cross-correlation analysis between DNA hypermethylation (ΔmC) in Dppa3KO ESCs and genomic occupancy of histone modifications and UHRF1 binding. Correlations are calculated over 1kb tiles. Positive correlations with ΔmC are bounded by a dashed-line square. **l,** Hypermethylation in Dppa3 KO ESCs is not associated with *Tet* downregulation. Expression of *Tet* genes in wt Dppa3KO ESC clones depicted as mRNA levels relative to *Gapdh*. Error bars indicate mean ± SD calculated from technical triplicate reactions from *n* = 2 biological replicates per genotype depicted. **m,** Schematic representation of the pSBtet-D3 (pSBtet-3xFLAG-Dppa3-IRES-DsRed) cassette for the Sleeping Beauty transposition-mediated generation of doxycycline (Dox) inducible *Dppa3* ESC lines. Abbreviations: inverted terminal repeat (ITR), tetracycline response element plus minimal CMV (TCE), 3xFLAG tag (3xF), internal ribosomal entry site (IRES), polyA signal (pA), constitutive RPBSA promoter (RPBSA), reverse tetracycline-controlled transactivator (rtTA), self-cleaving peptide P2A (P2A), puromycin resistance (PuroR). **n,** Confirmation of DPPA3 protein induction as assessed by immunofluorescence in uninduced ESCs (-Dox) or after 24 h of doxycycline treatment (+Dox). To illustrate the increase in DPPA3 protein levels, the same acquisition settings used to detect DPPA3, including a long exposure, in uninduced cells were applied for detection of DPPA3 after induction (+Dox (long exp.); bottom panel) leading to a saturated signal. Shorter exposure settings were also applied to the induced cells (+Dox (short exp.); middle panel) to better resolve the localization of DPPA3 after induction. The staining was repeated in 3 biological replicates with similar results. Scale bar: 10 µm. **o,** *Dppa3* expression before induction and after 3 or 6 days of doxycycline treatment in pSBtet-D3 ESCs. mRNA levels of *Dppa3* are shown relative to those in uninduced wt J1 ESCs after normalization to *Gapdh*. Error bars indicate mean ± SD calculated from technical triplicate reactions from *n* = 3 biological replicates.

In **e, g and h,** hypermethylation is defined as a gain in 5mC compared to wt ESCs (q value < 0.05; absolute methylation difference > 20%) with q-values calculated using a two-sided Wald test followed by p-value adjustment using SLIM. Source data are provided as a Source Data file.

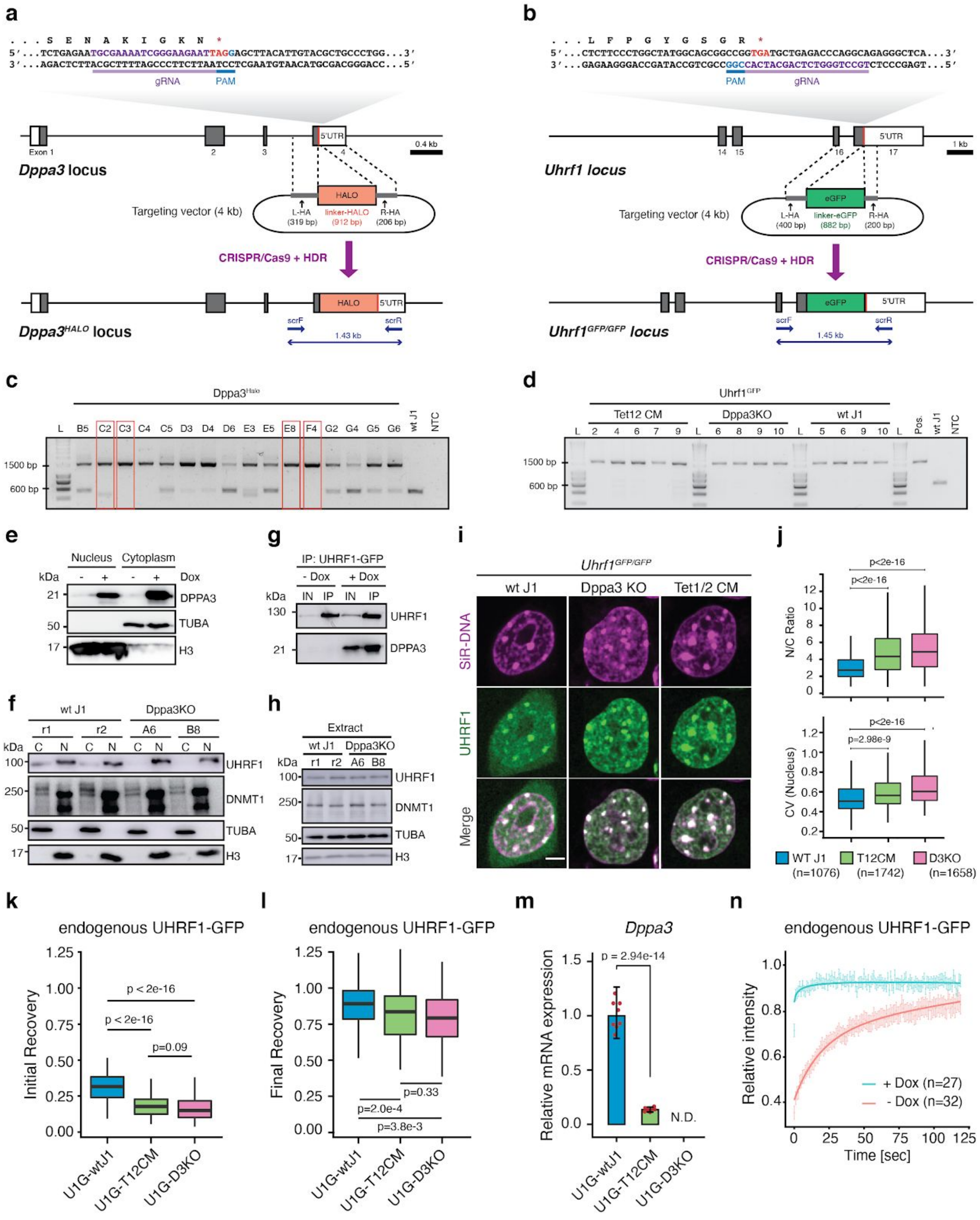

a
gRNA
PAM
Dppa3 locus
Targeting vector (4 kb)
HALO
L-HA (319 bp)
linker-HALO (912 bp)
R-HA (206 bp)
CRISPR/Cas9 + HDR
Dppa3HALO locus
1.43 kb
b
Uhrf1 locus
eGFP
L-HA (400 bp)
linker-eGFP (882 bp)
R-HA (200 bp)
Uhrf1GFP/GFP locus
1.45 kb
c
Dppa3Halo
d
Uhrf1GFP
Tet12 CM
Dppa3KO
wt J1
e
Nucleus
Cytoplasm
Dox
DPPA3
TUBA
H3
f
UHRF1
DNMT1
g
IP: UHRF1-GFP
h
Extract
i
Uhrf1GFP/GFP
SiR-DNA
Merge
j
N/C Ratio
CV (Nucleus)
WT J1 (n=1076)
T12CM (n=1742)
D3KO (n=1658)
k
endogenous UHRF1-GFP
Initial Recovery
l
Final Recovery
m
Dppa3
Relative mRNA expression
N.D.
n
Relative intensity
Time [sec]
+ Dox (n=27)
- Dox (n=32)

Supplementary Figure 4: TET proteins control UHRF1 chromatin association in mESCs by regulating DPPA3 levels

a,b, Schematic of the CRISPR/Cas9 targeting strategy used to insert fluorescent protein tags at the (**a**) *Dppa3* and (**b**) *Uhrf1* loci. Exons are depicted as boxes, with the coding sequences shaded in grey. Stop codons are indicated with a red line. The nucleotide and amino acid sequence encompassing the gRNA target site are enlarged for detail. gRNAs target sequences are colored purple and their respective PAMs blue. Donor constructs harboring the coding sequence for (**a**) HALO or (**b**) eGFP are flanked by homology arms (L-HA, left homology arm and R-HA, right homology arm), which are indicated with grey rectangles. Genotyping PCR primers used in (**c,d**) are shown as arrows along with the size of the amplicon confirming successful integration of the donor sequence. **c,d,** Results of genotyping PCRs for confirming the successful integration of (**c**) HALO into the *Dppa3* locus of wt J1 ESCs and (**d**) eGFP into the *Uhrf1* locus of wt J1, Dppa3KO, and T12CM ESCs. Wild-type (wt) and a negative control without DNA template (NTC) are depicted on the right. **c,** Clones with Sanger-sequence validated homozygous insertion of the HALO coding sequence are indicated with red boxes. **d,** PCR results after pre-screening via microscopy. All depicted clones have a correct homozygous insertion of eGFP as validated by Sanger-sequencing. PCRs were performed using the primers depicted in (**a,b**). **e,** DPPA3 is primarily but not exclusively localized to the cytoplasm in ESCs. Cytoplasmic and nuclear fractions from doxycycline (Dox) inducible FLAG-*Dppa3* ESC lines in the presence (+) or absence of Dox analyzed by immunoblot detection using the indicated antibodies or, in the case of DPPA3, an anti-FLAG antibody. **f,** DPPA3 regulates subcellular UHRF1 distribution. Cytoplasmic, "C", and nuclear, "N", fractions from wt and Dppa3KO ESCs analyzed by immunoblot detection using the indicated antibodies. The fractionation and immunoblot were repeated 2 times with similar results obtained. **g,** Immunoblot detection of anti-GFP immunoprecipitated material (IP) and the corresponding input (IN) extracts from U1G/wt + pSBtet-D3 ESCs treated before (-Dox) and after (+Dox) 24 h induction of *Dppa3* with doxycycline. Immunoblot was repeated 2 times with similar results obtained. **h,** UHRF1 and DNMT1 protein levels are unaffected by DPPA3 loss. Whole-cell extracts from wt and Dppa3KO ESCs analyzed by immunoblot detection using the indicated antibodies. Immunoblot depicts *n* = 2 biological replicates. **i,j,** DPPA3 alters the localization and nuclear patterning of endogenous UHRF1-GFP. **i,** Representative confocal images of UHRF1-GFP in live U1G-wt, U1G-T12CM, U1G-Dppa3KO ESCs with DNA counterstain (SiR-Hoechst). Scale bar: 5 µm. **j,** Quantification of endogenous UHRF1-GFP (top panel) nucleus to cytoplasm ratio (N/C Ratio) and (bottom panel) coefficient of variance (CV) within the nucleus of cells (number indicated in the plot) from *n* = 3 biological replicates per genotype. The assay was repeated 2 times with similar results. **k,l,** Endogenous DPPA3 prevents excessive UHRF1 chromatin binding in ESCs. Further analysis of the single cell FRAP data presented in Fig. 4c. showing the (**k**) initial and (**l**) final relative recovery of endogenous UHRF1-GFP. Intensity measurements 1 s and 60 s after photobleaching were used for calculating (**k**) initial recovery and (**l**) final recovery, respectively. **m,** *Dppa3* is significantly downregulated in U1G-T12CM ESCs. *Dppa3* transcript levels in U1G-wt, U1G-T12CM, U1G-Dppa3KO ESCs are depicted relative to mRNA levels in U1G-wt J1 ESCs after normalization to Gapdh. Error bars indicate mean ± SD calculated from technical triplicate reactions from *n* = 4 biological replicates (from n= 2 independent clones per genotype). N.D., expression not detectable. **n,** DPPA3 expression abolishes UHRF1 chromatin binding. FRAP analysis of endogenous UHRF1-GFP before (-Dox) and after 48 h of *Dppa3* induction (+Dox) in U1G-wt + pSBtet-D3 ESCs. The FRAP experiment was repeated at least 3 times with similar results obtained. In the boxplots in **j-l**, darker horizontal lines within boxes represent median values. The limits of the boxes indicate upper and lower quartiles, and whiskers extend to the most extreme value within 1.5 x the interquartile range from each hinge. P-values in **j-m**; Welch's two-sided t-test. In **n,** the mean fluorescence intensity of *n* cells (indicated in the plots) at each timepoint depicted as a shaded dot. Error bars indicate mean ± SEM. Curves (solid lines) indicate double-exponential functions fitted to the FRAP data. Source data are provided as a Source Data file.

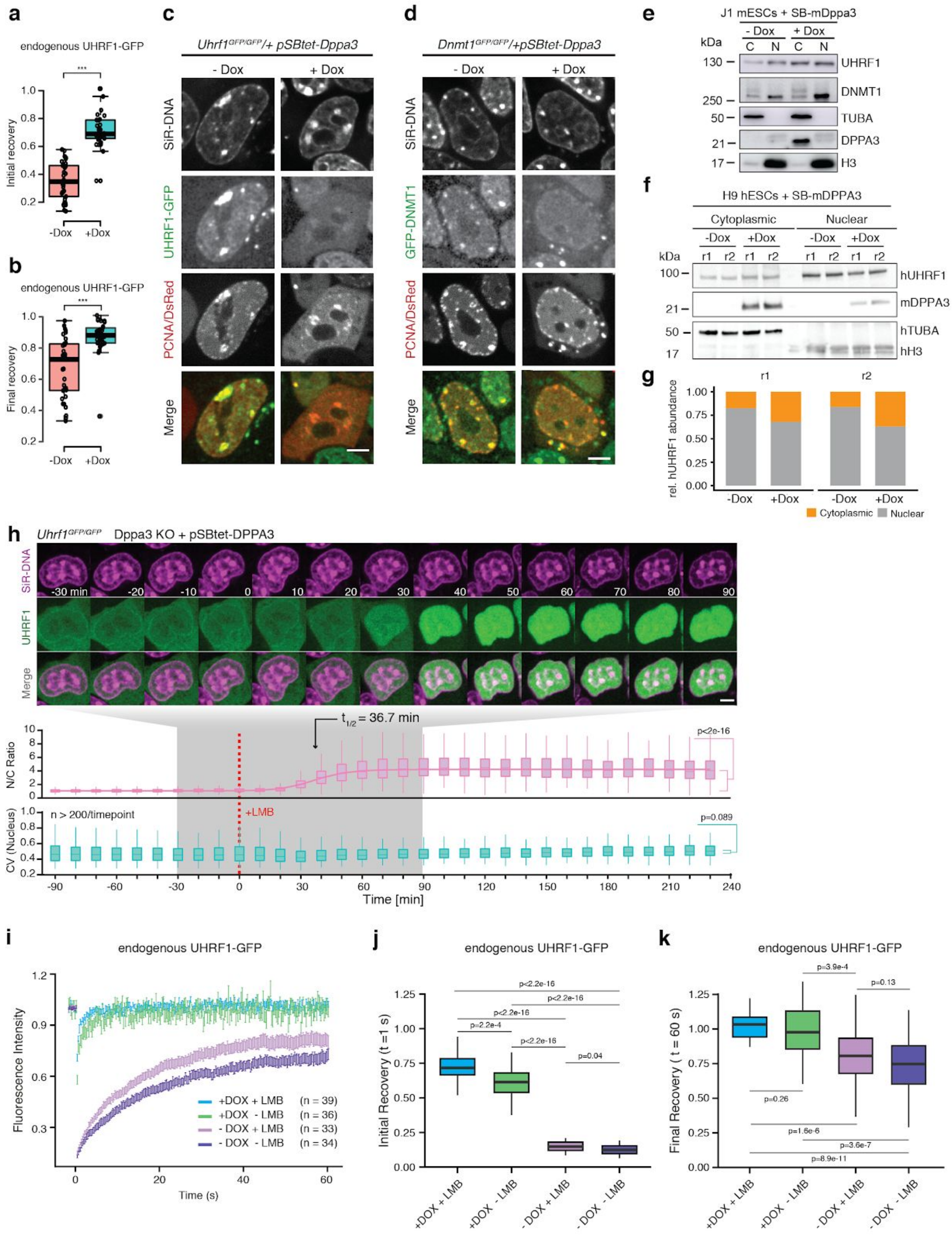
a
endogenous UHRF1-GFP
***
Initial recovery
-Dox
+Dox
b
endogenous UHRF1-GFP
***
Final recovery
-Dox
+Dox
c
Uhrf1GFP/GFP/+ pSBtet-Dppa3
- Dox
+ Dox
SiR-DNA
UHRF1-GFP
PCNA/DsRed
Merge
d
Dnmt1GFP/GFP/+pSBtet-Dppa3
- Dox
+ Dox
SiR-DNA
GFP-DNMT1
PCNA/DsRed
Merge
e
J1 mESCs + SB-mDppa3
- Dox
+ Dox
kDa
C
N
C
N
UHRF1
DNMT1
TUBA
DPPA3
H3
f
H9 hESCs + SB-mDPPA3
Cytoplasmic
Nuclear
-Dox
+Dox
-Dox
+Dox
kDa
r1
r2
hUHRF1
mDPPA3
hTUBA
hH3
g
r1
r2
rel. hUHRF1 abundance
-Dox
+Dox
-Dox
+Dox
Cytoplasmic
Nuclear
h
Uhrf1GFP/GFP Dppa3 KO + pSBtet-DPPA3
SiR-DNA
UHRF1
Merge
-30 min
t1/2 = 36.7 min
p<2e-16
N/C Ratio
n > 200/timepoint
+LMB
p=0.089
CV (Nucleus)
Time [min]
i
endogenous UHRF1-GFP
Fluorescence Intensity
+DOX + LMB (n = 39)
+DOX - LMB (n = 36)
- DOX + LMB (n = 33)
- DOX - LMB (n = 34)
Time (s)
j
endogenous UHRF1-GFP
Initial Recovery (t =1 s)
p<2.2e-16
p=2.2e-4
p=0.04
+DOX + LMB
+DOX - LMB
- DOX + LMB
- DOX - LMB
k
endogenous UHRF1-GFP
Final Recovery (t = 60 s)
p=3.9e-4
p=0.13
p=0.26
p=1.6e-6
p=3.6e-7
p=8.9e-11
+DOX + LMB
+DOX - LMB
- DOX + LMB
- DOX - LMB

Supplementary Figure 5: DPPA3 alters the localization and chromatin binding of endogenous UHRF1 and DNMT1 in ESCs

a,b, DPPA3 releases UHRF1 from chromatin. Initial (1 s post-bleach) (**a**) and final (120 s post-bleach) (**b**) relative recovery of UHRF1-GFP in *Uhrf1$^{GFP/GFP}$/SBtet-3xFLAG-Dppa3* ESCs before (-Dox) and after 48 h of *Dppa3* induction (+Dox) (+Dox: *n* = 27; -Dox: *n* = 32) (data from FRAP experiment in Supplementary Fig. 4m). **c,d,** DPPA3 disrupts the recruitment of UHRF1 and DNMT1 to replication foci. Live cell imaging illustrating the late S-phase localization of (**c**) UHRF1-GFP in U1G/wt + pSBtet-D3 ESCs or (**d**) GFP-DNMT1 in live *Dnmt1$^{GFP/GFP}$* + pSBtet-D3 ESCs before (-Dox) and after 48 h of *Dppa3* induction. Transfected RFP-PCNA marks sites of active DNA replication within the nucleus. In both c and d, expressed free DsRed is a marker of doxycycline induction (see cytoplasmic signal in +Dox PCNA/DsRed panels and Supplementary Fig. 3m). DNA was stained in live cells using a 30 min treatment of SiR-DNA (SiR-Hoechst). Data are representative of n = 2 independent experiments. Scale bar: 5 µm. **e-g,** DPPA3 alters the subcellular distribution of endogenous UHRF1 in mouse ESCs and human ESCs. **e,f,** Immunoblots of nuclear, "N", and cytoplasmic, "C", fractions from (**e**) U1G/wt + pSBtet-D3 mouse ESCs and (**f**) Human H9 ESCs + SBtet-3xFLAG-Dppa3 before (-Dox) and after 24 hours of *Dppa3* induction (+Dox) using the indicated antibodies. An anti-FLAG antibody was used for detection of FLAG-DPPA3. **g,** Quantification of the relative abundance of hUHRF1 in the cytoplasmic and nuclear fractions shown in (**f**) with the results of two independent biological replicates (r1 and r2) displayed. **h,** Nuclear export is dispensable for DPPA3-mediated inhibition of UHRF1 chromatin association. Localization dynamics of endogenous UHRF1-GFP in response to inhibition of nuclear export using leptomycin-B (LMB) after *Dppa3* induction in U1G/D3KO + pSBtet-D3 ESCs with confocal time-lapse imaging over 5.5 h (10 min intervals). *t = 0* corresponds to start of nuclear export inhibition (+LMB). (*top panel*) Representative images of UHRF1-GFP and DNA (SiR-Hoechst stain) throughout confocal time-lapse imaging. Scale bar: 5 µm. (middle panel) Nucleus to cytoplasm ratio (N/C Ratio) of endogenous UHRF1-GFP signal. (bottom panel) Coefficient of variance (CV) of endogenous UHRF1-GFP intensity in the nucleus. (*middle and bottom panel*) N/C Ratio and CV values: measurements in *n* > 200 single cells per time point (precise values can be found in the Source Data file), acquired at *n* = 20 separate positions . Curves represent fits of four parameter logistic (4PL) functions to the N/C Ratio (pink line) and CV (green line) data. The experiment was repeated 2 times with similar results. **i-k,** Nuclear export is not only dispensable for but attenuates DPPA3-mediated inhibition of UHRF1-GFP chromatin binding. **i,** FRAP analysis of endogenous UHRF1-GFP within the nucleus of U1G/D3KO + pSBtet-D3 ESCs before (-Dox) and after 48 h of *Dppa3* induction and before (-LMB) and after (+LMB) 3 h of leptomycin-B (LMB) treatment. The mean fluorescence intensity of *n* cells (indicated in the plots) at each timepoint is depicted as a shaded dot. Error bars indicate mean ± SEM. The FRAP experiment was repeated 3 times with similar results. **j,k,** Boxplots showing the (**j**) initial (0.25 s post-bleach) and (**k**) final (57.75 s post-bleach) relative recovery of endogenous UHRF1-GFP in the FRAP experiment plotted in **(i).** For each condition, recovery intensity was calculated from the same n cells depicted in **(i).**

For the boxplots in **a, b, h, j, k,** darker horizontal lines within boxes represent median values. The limits of the boxes indicate upper and lower quartiles, and whiskers extend to the most extreme value within 1.5 x the interquartile range from each hinge. All p-values were calculated using Welch's two-sided t-test and are depicted in the figure or as follows *** P < 2e-16. Source data are provided as a Source Data file.

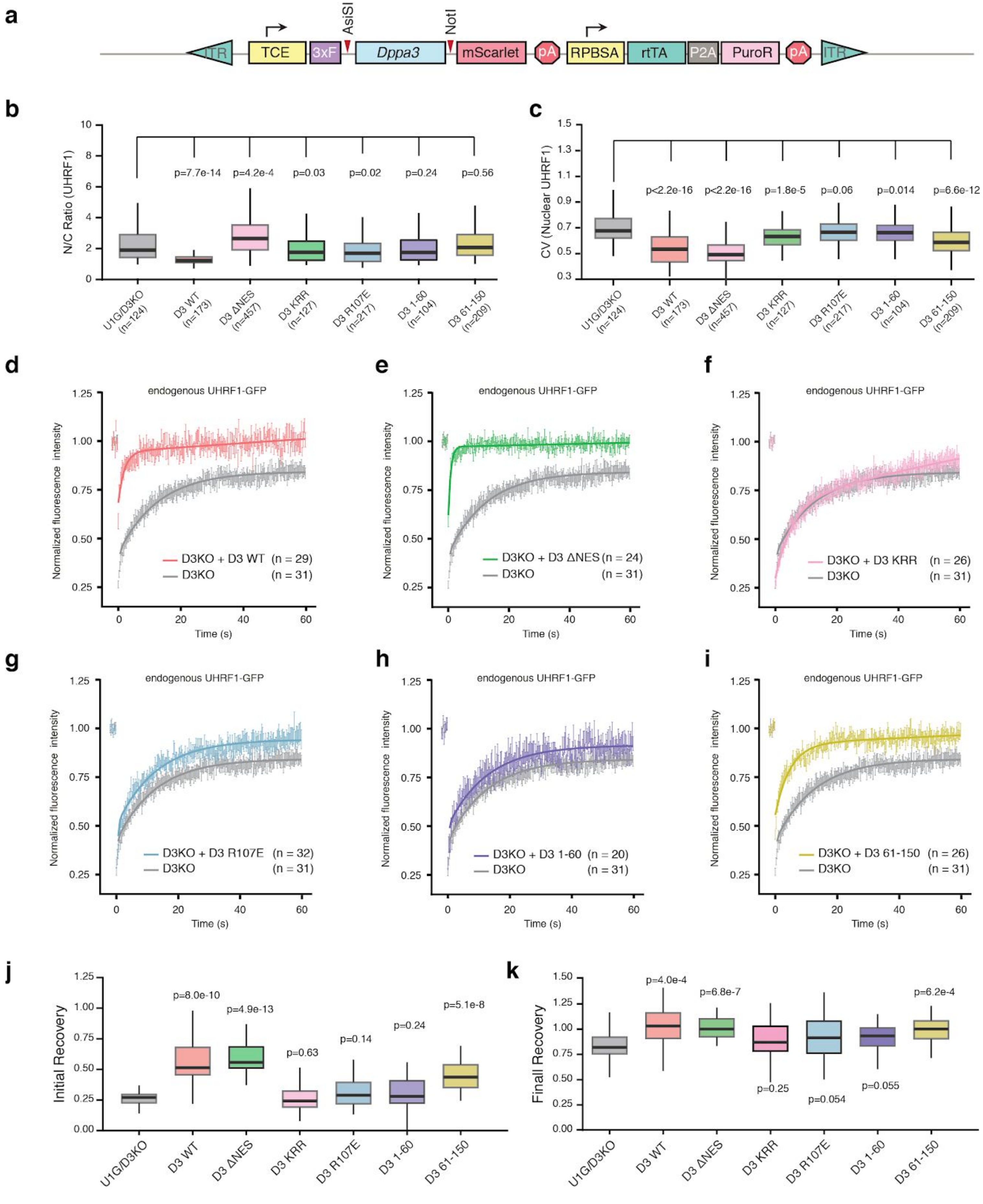
a
ITR
TCE
3xF
AsiSI
Dppa3
NotI
mScarlet
pA
RPBSA
rtTA
P2A
PuroR
pA
ITR
b
N/C Ratio (UHRF1)
p=7.7e-14
p=4.2e-4
p=0.03
p=0.02
p=0.24
p=0.56
U1G/D3KO (n=124)
D3 WT (n=173)
D3 ΔNES (n=457)
D3 KRR (n=127)
D3 R107E (n=217)
D3 1-60 (n=104)
D3 61-150 (n=209)
c
CV (Nuclear UHRF1)
p<2.2e-16
p<2.2e-16
p=1.8e-5
p=0.06
p=0.014
p=6.6e-12
d
endogenous UHRF1-GFP
Normalized fluorescence intensity
Time (s)
D3KO + D3 WT (n = 29)
D3KO (n = 31)
e
D3KO + D3 ΔNES (n = 24)
D3KO (n = 31)
f
D3KO + D3 KRR (n = 26)
D3KO (n = 31)
g
D3KO + D3 R107E (n = 32)
D3KO (n = 31)
h
D3KO + D3 1-60 (n = 20)
D3KO (n = 31)
i
D3KO + D3 61-150 (n = 26)
D3KO (n = 31)
j
Initial Recovery
p=8.0e-10
p=4.9e-13
p=0.63
p=0.14
p=0.24
p=5.1e-8
U1G/D3KO
D3 WT
D3 ΔNES
D3 KRR
D3 R107E
D3 1-60
D3 61-150
k
Finall Recovery
p=4.0e-4
p=6.8e-7
p=0.25
p=0.054
p=0.055
p=6.2e-4

Supplementary Figure 6: Further analysis of endogenous UHRF1-GFP localization and chromatin binding in response to mutant forms of mDPPA3

a, Schematic representation of the pSBtet-D3-mSC (pSBtet-3xFLAG-Dppa3-mScarlet) cassette for the Sleeping Beauty transposition-mediated generation of ESC lines with doxycycline (Dox) inducible expression of DPPA3-mScarlet fusions. Abbreviations: inverted terminal repeat (ITR), tetracycline response element plus minimal CMV (TCE), 3xFLAG tag (3xF), internal ribosomal entry site (IRES), polyA signal (pA), constitutive RPBSA promoter (RPBSA), reverse tetracycline-controlled transactivator (rtTA), self-cleaving peptide P2A (P2A), puromycin resistance (PuroR). Restriction sites used for exchanging the *Dppa3* coding sequences are indicated with red arrows. **b,c,** DPPA3 alters the nuclear localization of UHRF1 independently of promoting UHRF1 nucleocytoplasmic translocation. Quantification of endogenous UHRF1-GFP (**b**) nucleus to cytoplasm ratio (N/C Ratio) and (**c**) coefficient of variance (CV) within the nucleus of U1G/D3KO + pSBtet-D3 ESCs expressing the indicated mutant forms of *Dppa3*. The data depicted for each mutant in (b) and (c) was derived from analysis of the same *n* cells (indicated in the plot). **d-i** Nuclear export and the N-terminus of DPPA3 are dispensable for its inhibition of UHRF1 chromatin binding. FRAP analysis of endogenous UHRF1-GFP within the nucleus of U1G/D3KO + pSBtet-D3 ESCs expressing the following forms of DPPA3: (**d**) wild-type (D3 WT), (**e**) nuclear export mutant L44A/L46A (D3-ΔNES), (**f**) K85E/R85E/K87E mutant (D3-KRR), (**g**) R107E mutant (D3-R107E), (**h**) N-terminal 1-60 fragment (D3 1-60), (**i**) C-terminal 61-150 fragment (D3 61-150). In **d-i,** the mean fluorescence intensity of *n* cells (indicated in the plots) at each timepoint is depicted as a shaded dot. Error bars indicate mean ± SEM. Curves (solid lines) indicate double-exponential functions fitted to the FRAP data. Individual same fits correspond to those in Fig. 5c. FRAP of UHRF1 in cells expressing *Dppa3* mutants was repeated 2 times. **j,k,** Boxplots showing the (**j**) initial (0.25 s post-bleach) and (**k**) final (57.75 s post-bleach) relative recovery of endogenous UHRF1-GFP in the FRAP experiments plotted in **(d-i).** For each mutant, recovery intensity was calculated from the same n cells depicted in the corresponding plots **(d-i).**

In the boxplots in **b, c, j,** and **k,** darker horizontal lines within boxes represent median values. The limits of the boxes indicate upper and lower quartiles, and whiskers extend to the most extreme value within 1.5 x the interquartile range from each hinge. P-values were calculated using Welch's two-sided t-tests. Source data are provided as a Source Data file.

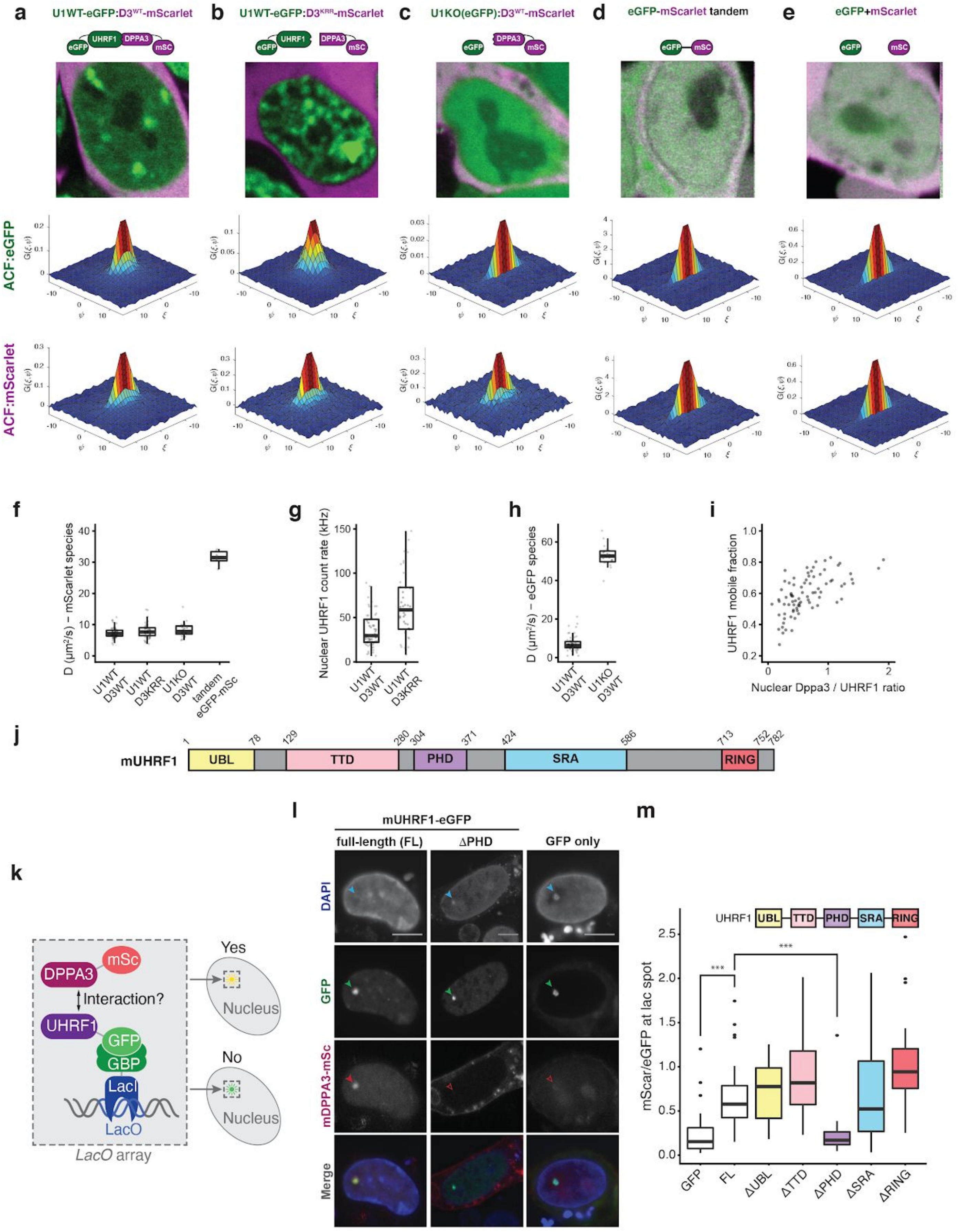
a
U1WT-eGFP:D3WT-mScarlet
b
U1WT-eGFP:D3KRR-mScarlet
c
U1KO(eGFP):D3WT-mScarlet
d
eGFP-mScarlet tandem
e
eGFP+mScarlet
eGFP
UHRF1
DPPA3
mSC
ACF:eGFP
ACF:mScarlet
f
D (μm²/s) – mScarlet species
U1WT D3WT
U1WT D3KRR
U1KO D3WT
tandem eGFP-mSc
g
Nuclear UHRF1 count rate (kHz)
U1WT D3WT
U1WT D3KRR
h
D (μm²/s) – eGFP species
U1WT D3WT
U1KO D3WT
i
UHRF1 mobile fraction
Nuclear Dppa3 / UHRF1 ratio
j
mUHRF1
UBL
TTD
PHD
SRA
RING
1
78
129
280
304
371
424
586
713
752
782
k
DPPA3
mSc
Interaction?
UHRF1
GFP
GBP
LacI
LacO
LacO array
Yes
No
Nucleus
l
mUHRF1-eGFP
full-length (FL)
ΔPHD
GFP only
DAPI
GFP
mDPPA3-mSc
Merge
m
UHRF1
mScar/eGFP at lac spot
GFP
FL
ΔUBL
ΔTTD
ΔPHD
ΔSRA
ΔRING
***

Supplementary Fig. 7: DPPA3 binds UHRF1 via its PHD domain to form a mobile complex in ESCs

a-e, Representative dual-color live-cell confocal images (top row) and associated 3D plots of the mean spatial autocorrelation (SACF) values of eGFP- (middle row) and mScarlet-labeled (bottom row) species in U1G/D3KO + pSBtet-D3 ESCs expressing the following forms of DPPA3-mScalet: (**a**) wild-type (U1WT:D3^{WT}) and (**b**) K85E/R85E/K87E mutant (U1WT:D3^{KRR}), in (**c**) Uhrf1KO ESCs expressing free eGFP and wild-type *Dppa3*-mScarlet (U1KO:D3^{WT}), and in control ESCs expressing (**d**) an eGFP-mScarlet tandem fusion (eGFP-mScarlet) and (**e**) free eGFP and free mScarlet (eGFP + mScarlet). In **a-e**, each image is the result of merging the sum projections of 250-frame image series acquired simultaneously from the eGFP (green) and mScarlet (magenta) channels. Autocorrelation plots are color-coded to indicate the mean correlation value. The spatial lag in the x-dimension (sensitive to fast timescales) is indicated by ξ, and the spatial lag in the y-dimension (sensitive to slower timescales) is indicated by ψ. **f,** The calculated diffusion coefficient of the mScarlet species in different cell types (those shown in (**a-d**)), derived from a two-component fit of mScarlet autocorrelation functions. Data are pooled from three independent experiments, except for the tandem eGFP-mScarlet, which was from two independent experiments. **g,** Photon count rates of UHRF1-eGFP in the nuclei of U1G/D3KOs expressing either DPPA3-WT (U1WT:D3^{WT}) or the DPPA3-KRR mutant (U1WT:D3^{KRR}). Data are pooled from three independent experiments. **h,** Calculated diffusion coefficients of UHRF1-GFP (in U1WT:D3^{WT}) or free eGFP (in U1KO:D3^{WT}), derived as those in C. Data are pooled from three independent experiments. **i,** A scatter plot showing the relationship between the mobile fraction of UHRF1-eGFP and the ratio of DPPA3-mScarlet:UHRF1-eGFP photon count rates in the nucleus. Data are pooled from three independent experiments. **j,** A schematic diagram illustrating the domains of mUHRF1: ubiquitin-like (UBL), tandem tudor (TTD), plant homeodomain (PHD), SET-and-RING-associated (SRA), and really interesting new gene (RING). **k,** Overview of the F3H assay used to find the domain of UHRF1 that binds to DPPA3. **l,m,** The PHD domain of UHRF1 is necessary for mediating the interaction with DPPA3. **l,** Representative confocal images of full-length UHRF1-GFP, ΔPHD UHRF1-GFP, and free GFP constructs (**j**) immobilized at the lacO array (indicated with green arrows). Efficient or failed recruitment of DPPA3-mScarlet to the lacO spot are indicated by solid or unfilled red arrows respectively. Similar results were obtained from n = 2 independent experiments. Scale bar: 5 µm. **m,** The efficiency of DPPA3-mScarlet recruitment to different UHRF1-eGFP deletion constructs immobilized at the lacO array is given as the fluorescence intensity ratio of mScarlet (DPPA3) to eGFP (UHRF1 constructs) at the nuclear LacO spot. Data represent the results of *n* = 25 cells analyzed from two independent experiments for each construct. P-values were calculated using a two-sided Welch's t-test: : *** P < 0.2e-16. In **f-h**, each data point indicates the measured and fit values from a single cell. In the box plots **f-h** and **m**, darker horizontal lines within boxes represent median values. The limits of the boxes indicate upper and lower quartiles, and whiskers extend to the most extreme value within 1.5 x the interquartile range from each hinge. Source data are provided as a Source Data file.

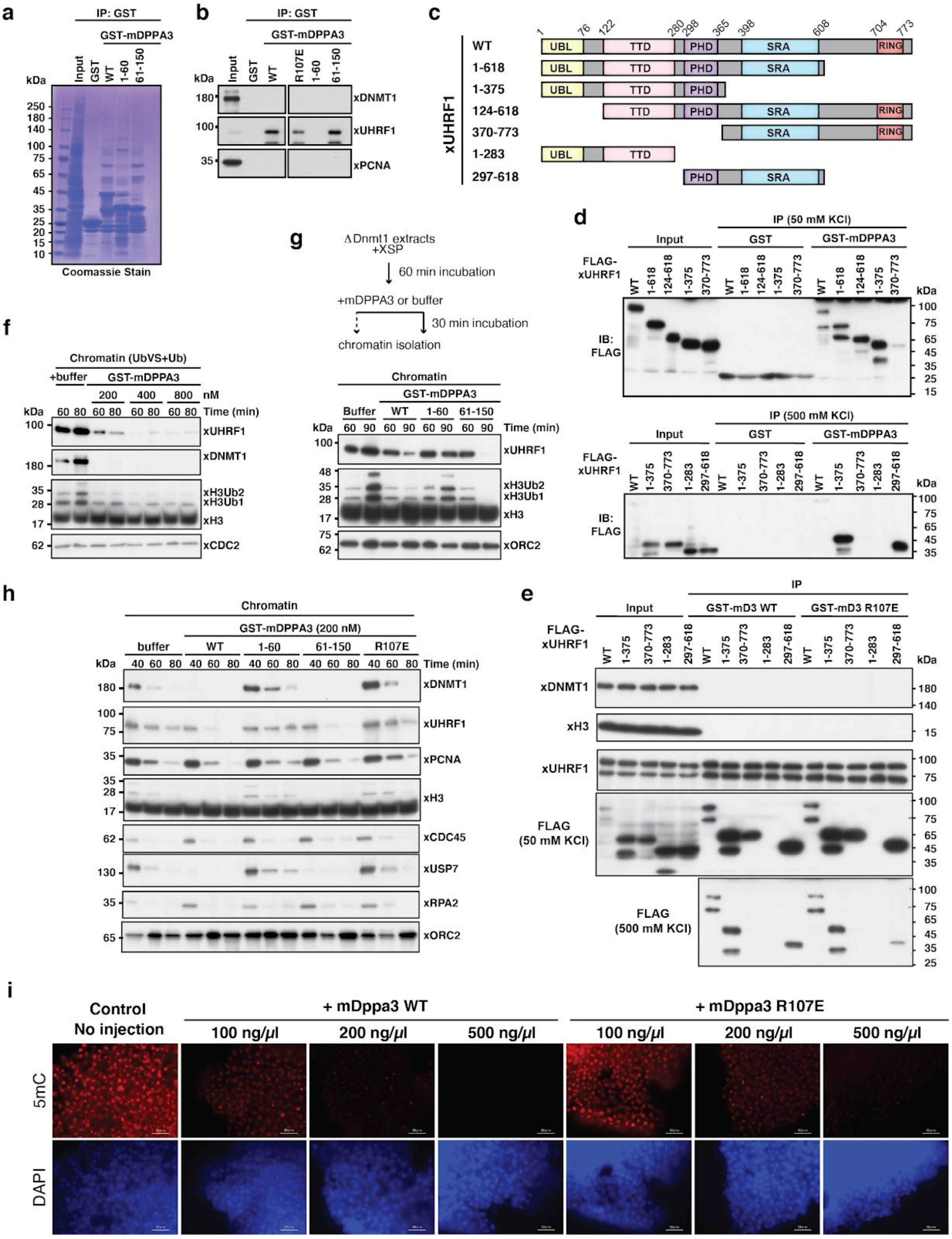
a
IP: GST
GST-mDPPA3
Input GST WT 1-60 61-150
kDa
Coomassie Stain
b
IP: GST
GST-mDPPA3
Input GST WT R107E 1-60 61-150
xDNMT1
xUHRF1
xPCNA
c
xUHRF1
WT
1-618
1-375
124-618
370-773
1-283
297-618
UBL
TTD
PHD
SRA
RING
d
IP (50 mM KCl)
Input
GST
GST-mDPPA3
FLAG-xUHRF1
IB: FLAG
IP (500 mM KCl)
e
IP
GST-mD3 WT
GST-mD3 R107E
xDNMT1
xH3
xUHRF1
FLAG (50 mM KCl)
FLAG (500 mM KCl)
f
Chromatin (UbVS+Ub)
+buffer
GST-mDPPA3
nM
Time (min)
xUHRF1
xDNMT1
xH3Ub2
xH3Ub1
xH3
xCDC2
g
ΔDnmt1 extracts +XSP
60 min incubation
+mDPPA3 or buffer
30 min incubation
chromatin isolation
Chromatin
GST-mDPPA3
Buffer
xORC2
h
Chromatin
GST-mDPPA3 (200 nM)
buffer
xPCNA
xCDC45
xUSP7
xRPA2
i
Control No injection
+ mDppa3 WT
+ mDppa3 R107E
100 ng/µl
200 ng/µl
500 ng/µl
5mC
DAPI

Supplementary Fig. 8: DPPA3 evolved in boreoeutherian mammals but its function is transferable to lower vertebrates

a, mDPPA3 C-terminus is sufficient for xUHRF1 binding. GST-tagged mDPPA3 wild-type (WT) and truncations (1-60 and 61-150) were immobilized on GSH beads and incubated with *Xenopus* egg extracts. The samples shown in Fig. 7c were subjected to SDS-PAGE and stained with Coomassie Blue. Representative of *n* = 3 independent experiments. **b,** High affinity interaction between mDPPA3 and xUHRF1 is weakened by R107E. GST-tagged mDPPA3 wild-type (WT), the point mutant R107E, and truncations (1-60 and 61-150) were immobilized on GSH beads and incubated with *Xenopus* egg extracts . Pull-downs were subjected to stringent 500 mM KCl washing. Bound proteins were analyzed using the indicated antibodies. Representative of *n* = 3 independent experiments. **c,** Schematic diagram illustrating the xUHRF1 deletion constructs used in the pull-downs in (**d,e**). **d,e,** xUHRF1 binds mDPPA3 via its PHD domain. *In vitro* translated xUHRF1 fragments were added to interphase *Xenopus* egg extracts. (**d**) GST and GST-mDPPA3 wild-type (WT) or (**e**) GST-tagged mDPPA3 wild-type (WT) and GST-tagged mDPPA3 R107E were immobilized on GSH beads and then used for GST-pulldowns on egg extracts containing the indicated recombinant xUHRF1 fragments. Bound proteins were analyzed using the denoted antibodies. Pull-downs were subjected to either 50 mM KCl or more stringent 500 mM KCl washing as indicated. Blots in d and e are representative of *n* = 3 independent experiments. **f,** mDPPA3 disrupts xUHRF1-dependent ubiquitylation of H3. As dual-monoubiquitylation of H3 (H3Ub2) is hard to detect given its quick turnover[7], we specifically enhanced ubiquitylation by simultaneous treatment of extracts with ubiquitin vinyl sulfone (UbVS), a pan-deubiquitylation enzyme inhibitor[8] and free ubiquitin (+Ub) as described previously[7]. Buffer or the displayed concentration of recombinant mDPPA3 were then added to the extracts. After the indicated times of incubation, chromatin fractions were isolated and subjected to immunoblotting using the antibodies indicated.Representative of *n* = 2 independent experiments. **g,** mDPPA3 displaces chromatin-bound xUHRF1. To stimulate xUHRF1 accumulation on hemi-methylated chromatin, *Xenopus* extracts were first immuno-depleted of xDNMT1 as described previously[9]. After addition of sperm chromatin, extracts were incubated for 60 min to allow the accumulation of xUHRF1 on chromatin during S-phase and then the indicated form of recombinant mDPPA3 (or buffer) was added. Chromatin fractions were isolated either immediately (60 min) or after an additional 30 min incubation (90 min) and subjected to immunoblotting using the antibodies indicated. Representative of *n* = 2 independent experiments. **h,** The region 61-150 of mDPPA3 is sufficient but requires R107 to inhibit xUHRF1 chromatin binding. Sperm chromatin was incubated with interphase *Xenopus* egg extracts supplemented with buffer (+buffer) and GST-mDPPA3 wild-type (WT), the point mutants R107E, truncations (indicated). Chromatin fractions were isolated after the indicated incubation time and subjected to immunoblotting using the antibodies indicated. Representative of *n* = 2 independent experiments. **i,** mDPPA3-mediated DNA demethylation in medaka. Representative 5mC immunostainings in late blastula stage (~8 h after fertilization) medaka embryos injected with wild-type *mDppa3* (WT) or *mDppa3* R107E (R107E) at three different concentrations (100 ng/µl, 200 ng/µl, or 500 ng/µl). Scale bars represent 50 µm. DNA counterstain: DAPI,4′,6-diamidino-2-phenylindole. Source data are provided as a Source Data file.

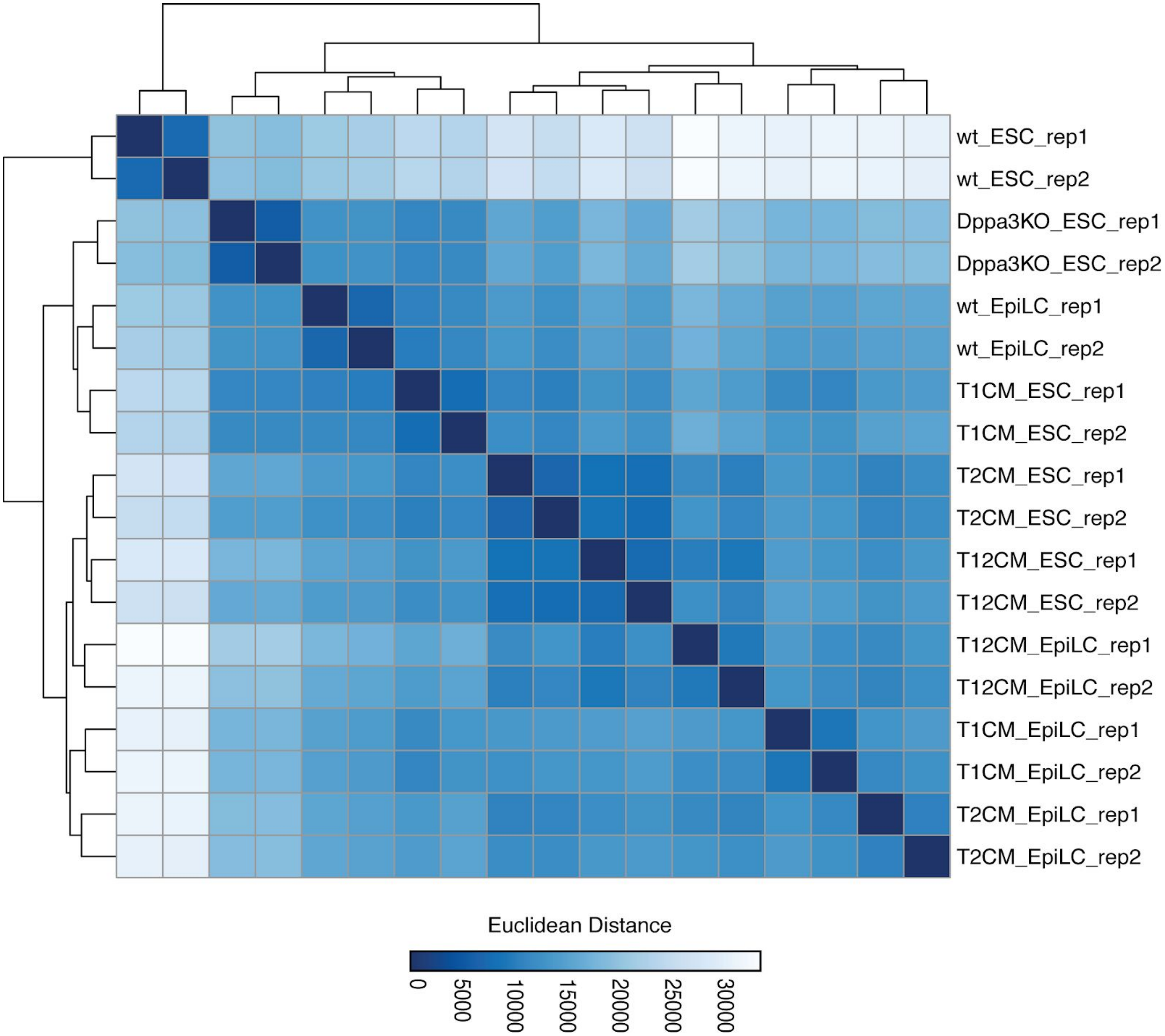

Supplementary Fig. 9: Methylome profiling of wild-type, T1CM, T2CM, T12CM, and Dppa3KO ESCs with RRBS

Heatmap depicting hierarchical clustering of the euclidean distance matrix generated from all RRBS samples used in this study.

References

1. von Meyenn, F. *et al.* Impairment of DNA Methylation Maintenance Is the Main Cause of Global Demethylation in Naive Embryonic Stem Cells. *Mol. Cell* **62**, 848–861 (2016).
2. Khoueiry, R. et al. Lineage-specific functions of TET1 in the postimplantation mouse embryo. *Nat. Genet.* **49**, 1061–1072 (2017).
3. Xiong, J. *et al.* Cooperative Action between SALL4A and TET Proteins in Stepwise Oxidation of 5-Methylcytosine. *Mol. Cell* **64**, 913–925 (2016).
4. Shen, L. *et al.* Genome-wide analysis reveals TET- and TDG-dependent 5-methylcytosine oxidation dynamics. *Cell* **153**, 692–706 (2013).
5. Mulholland, C. B. *et al.* Distinct and stage-specific contributions of TET1 and TET2 to stepwise cytosine oxidation in the transition from naive to primed pluripotency. *Sci. Rep.* **10**, 12066 (2020).
6. Sharif, J. *et al.* Activation of Endogenous Retroviruses in Dnmt1−/− ESCs Involves Disruption of SETDB1-Mediated Repression by NP95 Binding to Hemimethylated DNA. *Cell Stem Cell* **19**, 81–94 (2016).
7. Yamaguchi, L. *et al.* Usp7-dependent histone H3 deubiquitylation regulates maintenance of DNA methylation. *Sci. Rep.* **7**, 55 (2017).
8. Borodovsky, A. *et al.* A novel active site-directed probe specific for deubiquitylating enzymes reveals proteasome association of USP14. *EMBO J.* **20**, 5187–5196 (2001).
9. Nishiyama, A. *et al.* Uhrf1-dependent H3K23 ubiquitylation couples maintenance DNA methylation and replication. *Nature* **502**, 249–253 (2013).
