# Supplementary material for: Recent evolution of a TET-controlled and DPPA3/STELLA-driven pathway of passive DNA demethylation in mammals
Source: Nat Commun. 2020 Nov 24;11:5972. doi: 10.1038/s41467-020-19603-1 (PMC7686362; doi:10.1038/s41467-020-19603-1)
Supplement: Supplementary file 3 — Description of Additional Supplementary Files [file 41467_2020_19603_MOESM3_ESM.pdf]

Description of Additional Supplementary Files

File Name: Supplementary Data 1
Description: Differentially methylated promoters classified as either "TET-specific", "DPPA3-specific" or "common"

File Name: Supplementary Data 2
Description: Gene ontology analysis of TET-specific promoters

File Name: Supplementary Data 3
Description: Catalog of proteins interacting with FLAG-DPPA3 in ESCs

File Name: Supplementary Data 4
Description: Gene ontology analysis of significant DPPA3 interactors

File Name: Supplementary Data 5
Description: Oligonucleotides used in this study

File Name: Supplementary Data 6
Description: RRBS statistics
