# Supplementary material for: Recent evolution of a TET-controlled and DPPA3/STELLA-driven pathway of passive DNA demethylation in mammals
Source: Nat Commun. 2020 Nov 24;11:5972. doi: 10.1038/s41467-020-19603-1 (PMC7686362; doi:10.1038/s41467-020-19603-1)
Supplement: Supplementary file 10 — Reporting Summary [file 41467_2020_19603_MOESM10_ESM.pdf]

nature research

Corresponding author(s): *Double-blind peer review submissions: write DBPR and your manuscript number here instead of author names.*

Last updated by author(s): *YYYY-MM-DD*

Reporting Summary

Nature Research wishes to improve the reproducibility of the work that we publish. This form provides structure for consistency and transparency in reporting. For further information on Nature Research policies, see our Editorial Policies and the Editorial Policy Checklist.

Statistics

For all statistical analyses, confirm that the following items are present in the figure legend, table legend, main text, or Methods section.

n/a	Confirmed	
☐	☒	The exact sample size (*n*) for each experimental group/condition, given as a discrete number and unit of measurement
☐	☒	A statement on whether measurements were taken from distinct samples or whether the same sample was measured repeatedly
☐	☒	The statistical test(s) used AND whether they are one- or two-sided<br>*Only common tests should be described solely by name; describe more complex techniques in the Methods section.*
☒	☐	A description of all covariates tested
☒	☐	A description of any assumptions or corrections, such as tests of normality and adjustment for multiple comparisons
☐	☒	A full description of the statistical parameters including central tendency (e.g. means) or other basic estimates (e.g. regression coefficient) AND variation (e.g. standard deviation) or associated estimates of uncertainty (e.g. confidence intervals)
☐	☒	For null hypothesis testing, the test statistic (e.g. *F*, *t*, *r*) with confidence intervals, effect sizes, degrees of freedom and *P* value noted<br>*Give P values as exact values whenever suitable.*
☒	☐	For Bayesian analysis, information on the choice of priors and Markov chain Monte Carlo settings
☒	☐	For hierarchical and complex designs, identification of the appropriate level for tests and full reporting of outcomes
☒	☐	Estimates of effect sizes (e.g. Cohen's *d*, Pearson's *r*), indicating how they were calculated

Our web collection on statistics for biologists contains articles on many of the points above.

Software and code

Policy information about availability of computer code

Data collection	Real-time qPCR data was acquired using the LightCycler software (Roche). Live-cell imaging data was acquired with NIS Elements ver. 4.5 (Nikon). NIS Elements ver. 5.02.00 (Nikon) and Volocity (PerkinElmer) were used for acquiring FRAP data. RICS measurements were acquired using FABSurf (v 1.0). Immunoblot data was acquired using Amersham Imager 600 software (GE Healthcare). MST measurements were collected using Monolith NT.115 (NanoTemper Technologies). Microscopy data for the F3H assay were collected using Leica LAS AF. LC- MS/MS data was acquired on a Q Exactive HF-X Hybrid Quadrupole-Orbitrap Mass Spectrometer (Thermo Fisher Scientific) using Xcalibur 4.0 software (Thermo Fisher Scientific) and real time monitoring of the operational parameters was established by SprayQc software.<br>The PAM and MIA software is available as source code, requiring MATLAB, or as a precompiled, standalone distribution for Windows or MacOS at http://www.cup.uni-muenchen.de/ pc/lamb/software/pam.html or hosted in Git repositories under http://www.gitlab.com/PAM-PIE/PAM and http:// www.gitlab.com/PAM-PIE/PAMcompiled.
Data analysis	ive-cell imaging data was analyzed using CellProfiler Software (version 3.0). Fiji software (ImageJ 1.51j) was used to analyze images and create RGB stacks as well as perform analysis of FRAP and F3H data. Pulsed Interleaved Excitation Analysis with Matlab (v 7.10, PAM) software, Microtime Image Analysis (MIA) were used for RICS analysis. MST measurements were analyzed with MO. Affinity Analysis software version 2.1 (NanoTemper Technologies). Raw RRBS reads were first trimmed using Trim Galore (v.0.3.1) and aligned to the mouse genome (mm10) using bsmap (v.2.90) . CpG-methylation calls were extracted from the mapping output using bsmaps methratio.py in Python (v 3.6). The R package methylKit was used to identify differentially methylated regions. For analysis of public ChIP-seq data, reads were aligned to the mouse genome (mm10) with Bowtie (v.1.2.2). Peak calling and signal pile up was performed using MACS2 callpeak. Tag densities for promoters and 1kb Tiles were calculated using the deepTools2 computeMatrix module. RNA-seq libraries were processed and mapped to the mouse genome (mm10) using the zUMIs pipeline. UMI count tables were filtered for low counts using HTSFilter. Differential expression analysis was performed in R using DESeq2. Raw MS data was then analyzed with the MaxQuant software package (version 1.6.0.7). During the MaxQuant-based analysis the “Match between runs” option was enabled and the false discovery rate was set to 1% for both peptides (minimum length of 7 amino acids) and proteins. Relative protein amounts were determined by the MaxLFQ algorithm(Cox et al., 2014), with a minimum ratio count of two peptides. . For the downstream analysis of the MaxQuant output, the software Perseus (version 1.6.0.9) was

used. Statistical calculations were performed in R. Plots were generated using R Studio 1.2.1335 using ComplexHeatmap (v 1.17.1) and ggplot2 (v 3.3.0).

For manuscripts utilizing custom algorithms or software that are central to the research but not yet described in published literature, software must be made available to editors and reviewers. We strongly encourage code deposition in a community repository (e.g. GitHub). See the Nature Research guidelines for submitting code & software for further information.

Data

Policy information about availability of data

All manuscripts must include a data availability statement. This statement should provide the following information, where applicable:

- Accession codes, unique identifiers, or web links for publicly available datasets
- A list of figures that have associated raw data
- A description of any restrictions on data availability

Sequencing data reported in this paper are available at ArrayExpress (EMBL-EBI) under accessions E-MTAB-6785 [https://www.ebi.ac.uk/arrayexpress/experiments/E-MTAB-6785/](wild-type and Tet catalytic mutants RRBS), E-MTAB-6797 [https://www.ebi.ac.uk/arrayexpress/experiments/E-MTAB-6797/] (RNA-seq), E-MTAB-6800 [https://www.ebi.ac.uk/arrayexpress/experiments/E-MTAB-6800/](Dppa3KO RRBS), E-MTAB-9654 [https://www.ebi.ac.uk/arrayexpress/experiments/E-MTAB-9654/] (TaBA-seq of Tet catalytic mutants during Dppa3 induction) and E-MTAB-9653 [https://www.ebi.ac.uk/arrayexpress/experiments/E-MTAB-9653/] (TaBA-seq of Dppa3KO cells expressing Dppa3 mutant constructs). The raw mass spectrometry proteomics data from the FLAG-DPPA3 pulldown have been deposited at the ProteomeXchange Consortium via the PRIDE partner repository with the dataset identifier PXD019794. Publically available data sets used in this study can be found here: GSE77420 [https://www.ncbi.nlm.nih.gov/geo/query/acc.cgi?acc=GSE77420] (RRBS of TET triple knockout ESCs), GSE42616 [https://www.ncbi.nlm.nih.gov/geo/query/acc.cgi?acc=GSE42616] (PRDM14 ChIP-seq), GSE46111 [https://www.ncbi.nlm.nih.gov/geo/query/acc.cgi?acc=GSE46111] (5caC-DIP in TDK knockout ESCs), GSE57700 [https://www.ncbi.nlm.nih.gov/geo/query/acc.cgi?acc=GSE57700] (TET1 and TET2 ChIP-seq)

Field-specific reporting

Please select the one below that is the best fit for your research. If you are not sure, read the appropriate sections before making your selection.

☒ Life sciences ☐ Behavioural & social sciences ☐ Ecological, evolutionary & environmental sciences

For a reference copy of the document with all sections, see nature.com/documents/nr-reporting-summary-flat.pdf

Life sciences study design

All studies must disclose on these points even when the disclosure is negative.

Sample size	No statistical methods were used to predetermine sample size. Sample sizes were choses based on cost, experience and common sample sizes respective method.
Data exclusions	No data was excluded from the analysis
Replication	All the experimental findings were reliably reproduced in independent experiments as indicated in the Figure legends. The number of replicates used in each experiment are described in the Figure legends and/or in the Methods section, as are the Statistical tests used. P values values or adjusted P values are given where possible. Unless otherwise indicated, all statistical calculations were performed using R Studio 1.2.1335<br>Next-generation sequencing experiments include at least two independent biological replicates. RNA-seq experiments include n= 4 biological replicates comprised of n=2 independently cultured samples from 2 clones (for T1CM, T2CM, T12CM ESCs and EpiLCs) or 4 independently cultured samples (for wild-type ESCs and EpiLCs). For RRBS experiments, data are derived from n=2 biological replicates. For bisulfite sequencing of LINE-1 elements n=2 biological replicates were analyzed from 2 independent clones for T1CM, T2CM, T12CM, and Dppa3KO ESCs or 2 independent cultures for wt ESCs. LC-MS/MS quantification was performed on at least 4 biological replicates comprising at least 2 independently cultured samples (usually even more) from n=2 independent clones (T1CM, T2CM, T12CM, and Dppa3KO ESCs) or 4 independently cultured samples (wild-type ESCs).
Randomization	Xenopus frogs were selected randomly from our colony for ovulation. Medaka embryos were chosen randomly for injection. All other experiments were with performed using random samples as only using isogenic cell lines harvested at the same time to avoid biases and reduces covariates to a minimum.
Blinding	Blinding was not implemented in this study as in the overwhelming majority of experiments, analysis was inherently objective. For microscopy analysis, where possible, experimenter bias was avoided by selecting fields of view (or individual cells) for acquisition using the DNA stain (or another marker not being directly assessed in the experiment e.g. DsRed/mScarlet as a readout of Dppa3 induction or RFP-PCNA) to acquire data on UHRF1-GFP. To further reduce bias, imaging analysis was subsequently performed indiscriminately on all acquired images using semi-automated analysis pipelines (either with CellProfiler or Fiji scripts).

Reporting for specific materials, systems and methods

We require information from authors about some types of materials, experimental systems and methods used in many studies. Here, indicate whether each material, system or method listed is relevant to your study. If you are not sure if a list item applies to your research, read the appropriate section before selecting a response.

Materials & experimental systems

n/a	Involved in the study
☐	☒ Antibodies
☐	☒ Eukaryotic cell lines
☒	☐ Palaeontology and archaeology
☐	☒ Animals and other organisms
☒	☐ Human research participants
☒	☐ Clinical data
☒	☐ Dual use research of concern

Methods

n/a	Involved in the study
☒	☐ ChIP-seq
☒	☐ Flow cytometry
☒	☐ MRI-based neuroimaging

Antibodies

Antibodies used

rabbit anti-DPPA3 (polyclonal; 1:200; used in IF; Abcam, ab19878)
mouse anti-UHRF1 (monoclonal; 1:250; used in IF; Santa Cruz, sc373750)
goat anti-mouse A488 (polyclonal; 1:500; used in IF; Invitrogen, A11029)
donkey anti-rabbit Dylight594 (polyclonal; 1:500; used in IF; Dianova, 711-516-152)
anti-GFP-Booster ATTO488 (; 1:200; used in IF; Chromotek,)
mouse anti-5mC (monoclonal; 1:200; used in IF; Active Motif, 39649)
donkey anti-anti-rabbit A555 (polyclonal; 1:500; used in IF; Invitrogen, A31572)
donkey anti-anti-rabbit A488 (polyclonal; 1:500; used in IF; Dianova, 711-547-003)
rabbit anti-UHRF1 (polyclonal; 1:250; used in WB; Cell Signalling, D6G8E)
mouse anti-alpha-Tubulin (monoclonal; 1:500; used in WB; Sigma, T9026)
rabbit anti-H3 (polyclonal; 1:1000; used in WB; Abcam, ab1791)
mouse anti-GFP (monoclonal; 1:1000; used in WB; Roche,)
mouse anti-FLAG M2 (monoclonal; 1:1000; used in WB; Sigma, F3165)
rabbit anti-xDNMT1 (polyclonal; ; used in WB; , non-commercial)
rabbit anti-xDUHRF1 (polyclonal; ; used in WB; , non-commercial)
rabbit anti-USP7 (polyclonal; ; used in WB; Bethyl Lab., A300-033A)
rabbit anti-H3 (polyclonal; ; used in WB; Abcam, ab1791)
rat anti-TET1 (monoclonal; 1:10; used in WB; , non-commercial)
rat anti-alpha-Tubulin (monoclonal; 1:250; used in WB; Abcam, ab6160)
goat anti-rat HRP (polyclonal; 1:1000; used in WB; Jackson ImmunoResearch,)
goat anti-rabbit HRP (polyclonal; 1:1000; used in WB; BioRad,)
mouse anti-xCDC45 (monoclonal; ; used in WB; , non-commercial)
mouse anti-xRPA2 (monoclonal; ; used in WB; , non-commercial)
mouse anti-PCNA (monoclonal; ; used in WB; Santa Cruz, sc56)

Validation

rabbit anti-DPPA3 was validated for IF/WB (for details see https://www.abcam.com/stellar-antibody-ab19878.html)
mouse anti-UHRF1 was validated for IF/WB (for details see https://www.scbt.com/de/p/uhrf1-antibody-h-8)
mouse anti-5mC was validated for IF/WB (for details see https://www.activemotif.com/catalog/details/39649/5-methylcytosine-5-mc-antibody-mab-clone-33d3)
rabbit anti-UHRF1 was validated for WB (for details see https://www.cellsignal.com/products/primary-antibodies/uhrf1-d6g8e-rabbit-mab/12387?Ntk=Products&Ntt=12387)
mouse anti-alpha-Tubulin was validated for IF/WB (for details see https://www.sigmaaldrich.com/catalog/product/sigma/t9026)
rabbit anti-H3 was validated for WB (for details see https://www.abcam.com/histone-h3-antibody-nuclear-marker-and-chip-grade-ab1791.html)
mouse anti-GFP was validated for WB (for details see https://www.sigmaaldrich.com/catalog/product/roche/11814460001)
mouse anti-FLAG M2 was validated for WB/IF (for details see https://www.sigmaaldrich.com/catalog/product/sigma/f3165)
rabbit anti-xDNMT1 was validated for WB (for details see Nishiyama et al., 2013)
rabbit anti-xDUHRF1 was validated for WB (for details see Nishiyama et al., 2013)
rabbit anti-USP7 was validated for WB (for details see https://www.bethyl.com/product/A300-033A/USP7+Antibody)
rabbit anti-H3 was validated for WB (for details see https://www.abcam.com/histone-h3-antibody-nuclear-marker-and-chip-grade-ab1791.html)
rat anti-TET1 was validated for WB/IF (for details see Bauer et al., 2015)
rat anti-alpha-Tubulin was validated for WB/IF (for details see https://www.abcam.com/tubulin-antibody-yl12-loading-control-ab6160.html)
mouse anti-xCDC45 was validated for WB (for details see Françon et al., 2004)
mouse anti-xRPA2 was validated for WB (for details see Shintomi et al., 2009)
mouse anti-PCNA was validated for WB (for details see https://www.scbt.com/de/p/pcna-antibody-pc10)

Shintomi K, Hirano T. Releasing cohesin from chromosome arms in early mitosis: opposing actions of Wapl-Pds5 and Sgo1. Genes Dev 2009;23:2224–36.

Françon P, Lemaître J-M, Dreyer C, Maiorano D, Cuvier O, Méchali M. A hypophosphorylated form of RPA34 is a specific component of pre-replication centers. J Cell Sci 2004;117:4909–20.

Nishiyama A, Yamaguchi L, Sharif J, Johmura Y, Kawamura T, Nakanishi K, et al. Uhrf1-dependent H3K23 ubiquitylation couples maintenance DNA methylation and replication. Nature 2013;502:249–53.

Bauer C, Göbel K, Nagaraj N, Colantuoni C, Wang M, Müller U, et al. Phosphorylation of TET proteins is regulated via O-GlcNAcylation by the O-linked N-acetylglucosamine transferase (OGT). J Biol Chem 2015;290:4801–12.

Eukaryotic cell lines

Policy information about cell lines

Cell line source(s)	All mouse ESC lines used in this study are derived from wild-type J1 (129/SvJae strain) mouse embryonic stem cells were generated by Dr. Rudolf Jaenisch, Whitehead Institute (MIT). H9 human ESCs were acquired from Wicell (WB66593). Human embryonic kidney 293T (HEK293T) cells were acquired from the Leibniz Institute - German Collection of Microorganisms and Cell Cultures (DSMZ #ACC635; Braunschweig, GER). BHK cells were a gift from D. L. Spector.
Authentication	Gene edited cell lines were routinely authenticated by genotyping PCRs. STR authentication however was not performed on any cell line used in this study.
Mycoplasma contamination	Cell lines were routinely checked for mycoplasma contamination via PCR. All cell lines used in this study have been found to be negative for mycoplasma contamination.
Commonly misidentified lines (See ICLAC register)	Commonly misidentified cell lines were not used in this study

Animals and other organisms

Policy information about studies involving animals; ARRIVE guidelines recommended for reporting animal research

Laboratory animals	Xenopus laevis (males and females), mature, from 1 to 4 years old. Obtained from Kato-S-kagaku. Medaka (Oryzias latipes), males and females , d-rR strain was used. The age of Medaka (Oryzias latipes) embryos used was 18hpf (Fig. 7f andi) and 8hpf (7g,h, and Supp. 8i)
Wild animals	no wild animals were used in this study
Field-collected samples	no field-collected samples were used in this study
Ethics oversight	Medaka (Oryzias latipes) and Xenopus laevis were maintained and handled according to the animal care regulations at the University of Tokyo by the "Committee on Animal Care and Use of the Graduate School of Science".

Note that full information on the approval of the study protocol must also be provided in the manuscript.
